# Indigenous Subarctic Food Systems in Transition: Amino Acid Composition (Including Tryptophan) in Wild-Harvested and Processed Meats

**DOI:** 10.1155/2019/7096416

**Published:** 2019-06-27

**Authors:** Nicole Spiegelaar, Ian D. Martin, Leonard J. S. Tsuji

**Affiliations:** University of Toronto Scarborough, Toronto, Canada

## Abstract

Indigenous people of northern Canada traditionally lived a nomadic lifestyle subsisting on wild game and fish for thousands of years. With colonization came an increasing dependence on imported processed foods. This dietary change has often been reported to be one of the factors leading to Indigenous health and wellbeing disparities worldwide. We determined the amino acid (AA) profile including tryptophan (Trp) of wild meats (game and fish) and processed meats found in the traditional and modern diets of Indigenous subarctic communities in Canada. Trp is a limited essential AA necessary for synthesis of serotonin (5-HT), an important neurotransmitter and homeostatic regulator. The dietary ratio of Trp relative to other large neutral AAs (LNAA) can alter Trp transport and 5-HT synthesis in the brain. We determined AA composition of wild meats and processed meats using standardized NaOH and HCl hydrolysis for Trp and other AAs, respectively, followed by ultraperformance liquid chromatography. A Principal Components Analysis revealed that overall AA composition is significantly different between wild and processed meats. (M)ANOVA showed significantly higher protein in wild meats (wet weight, ww). Trp was significantly lower in processed meat samples (n=15; 0.18g/100g ± 0.02 ww) compared to wild meat samples (n=25; 0.24g/100g ± 0.06 ww). The proportion of Trp:LNAA and Trp in sample protein were not significantly different between wild (1:21-1:27, 0.92-1.27 g/100g protein) and processed (1:20-1:24, 1.03-1.27 g/100g protein) meats. Within wild meats, AA composition is significantly different between fish and waterfowl, fish and moose, and moose and goose. (M)ANOVA results indicate significantly higher protein in goose compared to moose and fish and in moose compared to fish. We compared our Trp findings to previous analyses and discuss the substantial gap in human nutritional studies of Trp.

## 1. Introduction

In the past century of cultural assimilation, Indigenous communities of northern Canada transitioned from a traditional high-protein diet of mammals, game birds, and fish to a modern diet dominated by highly processed commercial foods [1–5]. This dietary transition has recently been associated with a decline in mental health and a disproportionately high prevalence of diabetes among Indigenous people, especially in remote areas [6, 7]. Herein we consider the importance of postcolonial nutritional barriers to health and wellbeing. During interviews with Indigenous James Bay Cree of subarctic Ontario, Canada, participants described a shift from wild game and fish to processed meats and sugary foods in their lifetime; they associated this change with reduced mental and physical health and related wild meat consumption to feeling “full” and “happy.”

In the present study, we determined Trp, Trp:LNAA, and overall AA content in raw wild meats (traditional diet) and right-from-the-package processed meats (modern diet) from a subarctic Cree community in Ontario. We sought to determine if the dominant sources of protein in the modern diet are an adequate substitute for traditional wild meats in terms of Trp, Trp:LNAA, and AA composition, given the following facts and concerns: (i) the significant role of Trp in mood/metabolic regulation and adaptive response to stress [8–12], (ii) the high comorbidity of metabolic and mood disorders prevalent in subarctic Indigenous communities [13, 14], (iii) the unique susceptibility of Trp to degradation under conditions of industrial food processing [15–17], and (iv) the unique knowledge gap on Trp composition in the human diet, unlike other AAs.

Dietary Trp is essential to human cognitive, emotional, and energy function [10, 18] due to its role as the rate-limiting precursor to the neurotransmitter serotonin (5-HT) [19, 20]. Trp is one of several large neutral AAs (LNAA) that compete for transport across the blood-brain barrier, including the other aromatic AAs tyrosine and phenylalanine and the branched-chain AAs valine, isoleucine, and leucine [21]. Changes in dietary Trp:LNAA alter blood plasma Trp:LNAA proportionately, altering central Trp uptake and central 5-HT synthesis [20, 22–25].

Although Trp composition varies among dietary proteins [26–28], Trp composition literature is sparse due to the unique structure of the amino acid. Trp has an electron-rich and highly reactive indole ring that makes it vulnerable to degradation [15, 29]. This means that, along with cysteine, Trp is the most challenging and costly AA to recover and is frequently excluded from general AA composition studies [30, 31]. Determination of Trp content, digestibility, and absorption is much more common in animal nutrition studies because of the agroeconomic benefits of regulated dietary Trp in yield and quality [32, 33].

In 2011, the FAO (Food and Agricultural Organization) announced human dietary Trp requirements 2-3 times higher than previously reported in 1998. They also recognized AAs as individual nutrients rather than general constituents of protein quality [34]. The FAO has since requested an update of AA content within nutrient databases due to advances in quantification methods that were standardized in 2000 [34]. The labile nature of Trp is an added impetus for its quantification in the human diet. Research in the 1980s and early 1990s showed significant Trp loss from food exposed to severe heat in the presence of oxygen [15–17]. This loss was accelerated by the presence of a variety of food additives, preservatives, and chemicals associated with commercial food processing [15, 16, 29]. AA proportions may be disrupted by other techniques of processing, mechanical modifications, chemicals, and materials used in cleaning, storing, and packaging, and the addition of reducing sugars, starch, other nutrients and preservatives [16, 17, 29, 35, 36]. However, these findings are limited by the methods of the time and a research gap since then.

## 2. Methods 

### 2.1. Sample Collection

Two groups of food were collected from Fort Albany First Nation in the James Bay region: right-from-the-package processed meats, n=15 (Table 1), and raw wild meats, n=25 (Table 2). For the purpose of this baseline AA content study, we compare dominant AA sources of the traditional and modern Cree diets as the community acquires them. Both direct-from-the-package (or can) processed meats and wild meats typically undergo further heat processing by the end user prior to consumption in our study communities. The present study was not designed to compare or adjust for the effects of various home-cooking methods prior to end user consumption (e.g., frying, boiling, baking, smoking, roasting, microwaving, or otherwise heating).

Wild species were selected based on popularity determined through interviews with 24 community harvesters. Processed meats were selected based on purchase frequencies by the Fort Albany community at their two food supply stores. Within these two food sources, five types of processed meat (three replicates) and five species of wild meat (five replicates) were collected (Tables 1 and 2). Each processed sample was sourced from a different lot number and/or expiry date. A full ingredient list for processed foods can be found in Table 10. Each wild meat sample was of the same type—striated muscle because it comprises the largest portion of the total edible mass—and from a different specimen. Studies have shown variability in Trp composition between different tissues of a given species [37, 38] and between genetically distinct individuals of the same species [39, 40]. All samples were processed within 24 hours after being harvested and placed in a Ziploc bag, prior to being stored in a -20°C freezer. Frozen samples were shipped in coolers with ice packs to the laboratory for analyses.

### 2.2. Sample Processing

Samples were processed at the Institut de Recherche sur les Zones Côtières in Shippagan, New Brunswick, CA.

#### 2.2.1. Homogenization

Chicken nugget breading was removed to reduce potential Trp degradation. Samples were homogenized based on AOAC methods 937.07 (fish) and 983.18 (meat). 

#### 2.2.2. Fat Removal and Determination

To eliminate exposure to heat, samples were defatted using a method modified from Folch et al. [41]. Approximately 2-5g of each sample was homogenized in 10mL MeOH and 20mL CH_3_Cl using a polytron and combined with 40mL KCl 0.1M in a separatory funnel. The test tube used for the homogenization was rinsed twice with 10mL MeOH and 20mL CH_3_Cl. This solvent was added to the separatory funnel, vigorously mixed and left to rest. The bottom CH_3_Cl phase was filtered through anhydrous sodium sulfate and recovered. A second portion of 60mL of CH_3_Cl was added to the separatory funnel and mixed before being allowed to rest for phase separation. This second portion of CH_3_Cl was recovered with the first portion, CH_3_Cl was evaporated, and crude fat was recovered for weighing.

#### 2.2.3. Moisture Removal and Determination

Following Folch extraction, the upper phase of the mixture containing the defatted solid, MeOH, and KCl solution was left to rest. Solids were decanted and recovered for freeze-drying. Moisture content was determined from the original sample using a method modified from AOAC 950.46: 18 hours at 100-102°C.

#### 2.2.4. Protein Determination

Nitrogen content was determined in dry samples by the Kjeldahl method modified from AOAC 981.10, using a conversion factor of 6.25.

#### 2.2.5. “Other” Determination

“Other” (carbohydrate, ash, sodium, and preservatives) content was not directly determined, but indirectly estimated as the remaining dry sample after protein determination.

### 2.3. AA Analysis

AA composition was determined at the Hospital for Sick Children SPARC BioCentre, Toronto, Ontario, CA: Trp via NaOH hydrolysis and 18 other AAs via HCL hydrolysis [42]. Cysteine was not determined.

#### 2.3.1. Hydrolysis

For each sample, two weighed extractions (~0.0100g) of the defatted sample were transferred into two separate 8x40 mm borosilicate glass shell vials and underwent separate hydrolysis for Trp and the other AAs. For Trp analysis, the sample underwent hydrolysis with 225*μ*L of 4.2N NaOH and 50*μ*L of 25*μ*M/mL norleucine as internal standard for 20-24 hours at 110°C. The sample was centrifuged with 225*μ*L of 4.2N HCl for 5 minutes. For all other AAs, the sample was hydrolyzed with 450*μ*L of 6N HCl with 1% phenol and 50*μ*L of 25*μ*M/mL norleucine as internal standard for 48 hours at 110°C. After hydrolysis, each sample vial was centrifuged and an aliquot of 10*μ*L was transferred to a 6x55 mm borosilicate glass culture tube and dried using a vacuum centrifuge.

#### 2.3.2. Derivatization

After drying, each sample was treated with a redrying solution of methanol:water:triethylamine (2:2:1), vortex-mixed, and dried under vacuum for 15 minutes. The sample was derivatized for 20 minutes at room temperature with a derivatizing solution made up of methanol:water:triethylamine:phenylisothiocyanate (PITC) (7:1:1:1). The derivatizing solution was removed under vacuum for 15 minutes. The derivatized sample was again washed with the redrying solution, vortex-mixed, and dried under vacuum for 15 minutes.

#### 2.3.3. UPLC Analysis

AA analysis was performed on a Waters Acquity Ultra Performance Liquid Chromatography (UPLC) System. UPLC is comparable to HPLC with greater limits of detection [43]. The derivatized sample was dissolved in a given amount of sample diluent (pH 7.40) and an aliquot was injected into the column, running on a modified PICO-TAG gradient. Column temperature was at 48°C. The derivatized AAs were detected at 254 nm.

The Waters Acquity UPLC system employed consists of a Binary Solvent Manager, a Sample Manager, a TUV Detector, and a Waters Acquity UPLC BEH C18 column (2.1 X 100 mm). Data were collected, stored, and processed using Waters Empower 3 Chromatography software. Drying was done using a Tomy CC-181 Centrifugal Concentrator with a Sargent-Welch Model 8821 Vacuum pump. AA standards were purchased from Waters Corporation (Milford, MA, USA).

### 2.4. Statistical Analysis

Statistical analysis was performed on proximate composition (fat, moisture, protein, and “other”), Trp content, and Trp:LNAA. These data were inspected for outliers and miscodings before analysis.

**Figure 1:**
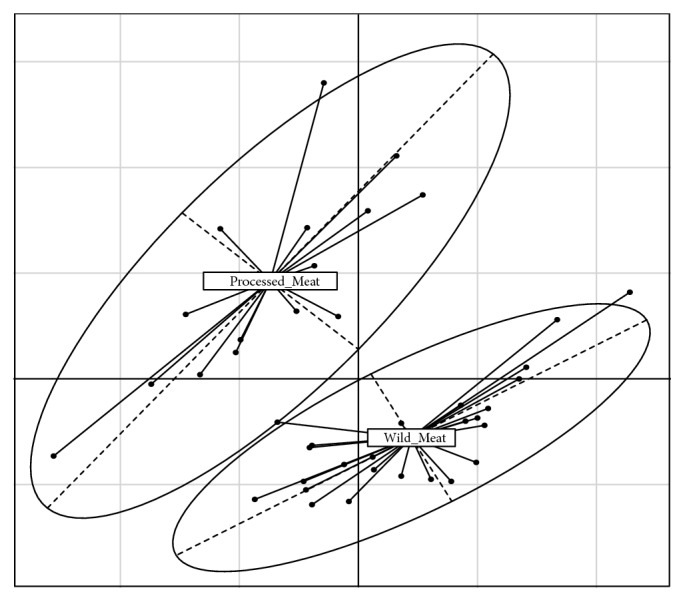
95% Confidence Ellipses of 19 AAs on PC-1 vs PC-2 Distinguish Processed and Wild Meat Sources.

**Figure 2:**
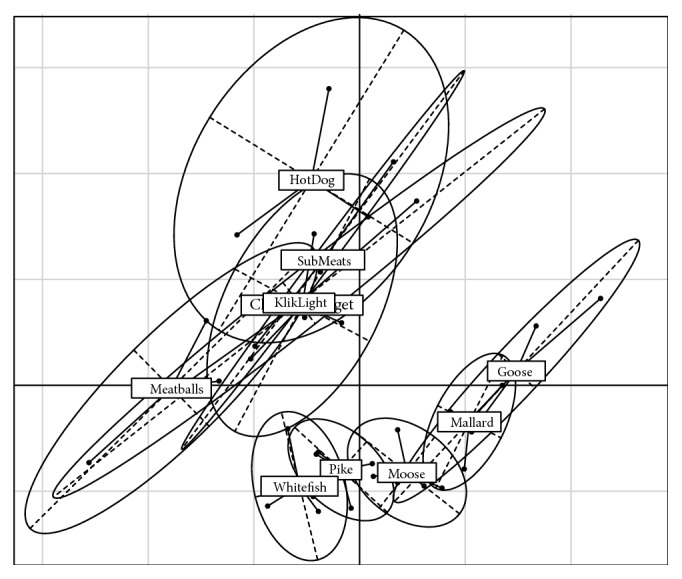
95% Confidence Ellipses of 19 AAs on PC-1 vs PC-2 Distinguish Processed ^a^ Meat Types and Wild Meat Types. a. The Klik Light label partially obscures the Chicken Nugget label.

We used Principal Components Analysis (PCA) to explore variation in the AA data. All 19 AA concentrations were transformed as log_10_ (1 + [concentration]) to reduce skewness and nonnormal distributions in data. The 19 AA variables were reduced to three composite variables using PCA (rigid rotation) of the correlation matrix of the original variables. We constructed graphical displays of covariance confidence regions for wild and processed meats at the 95% confidence level. These confidence ellipsoids show the composition of wild and processed meats in amino acid PC space (Figures 1 and 2).

We performed a 1-way (M)ANOVA to examine differences between processed and wild meats with the following variables: protein, moisture, fat, “other”, and tryptophan (raw values, log values, and in ratio to LNAA), as well as AA PC scores from the analysis above. Similarly, we performed 1-way (M)ANOVAs (with post hoc tests) to examine differences within processed meats and within wild meats with the same suite of variables.

Statistical analyses were carried out using SPSS v. 22. Ellipsoid plots were produced using ADE4 in R.

## 3. Results

Descriptive statistics are listed below for proximate nutritional composition (Table 3), total Trp content, ww (Table 4), Trp/LNAA in protein (Table 5), and PC loading scores for all 19 AAs (Table 6).

Trp as a percentage of protein ranged within the common estimate of 1-2% of protein for all samples except fish, which fell just below 1% (Table 4). Mallard, goose, Klik Light, and unbreaded chicken nuggets had the highest concentration of Trp, as a percentage of protein. Descriptive statistics for other AAs are in Table 5.

Overall MANOVA statistics showed highly significant differences between wild and processed meats. A 1-way ANOVA revealed significant differences between these groups for all measured variables, with the exception of Trp:LNAA ratio and AA PC-3 (Table 7). Wild meats had significantly higher concentrations of protein, moisture, and Trp and lower concentrations of fat and “other.”

Trp content on a g/100g ww-basis serving was significantly higher in wild meats (0.24%) than processed meats (0.18%) (Tables 4 and 7). Trp among wild samples was highest in waterfowl and lowest in fish.

MANOVA revealed significant differences within the wild meats group and within the processed meats group. One-way ANOVAs revealed significant differences within commercial meats for some variables (moisture, fat, “other,” and AA PC-3), while, within the wild meats, significant differences were noted for all measured variables, with the exception of “other” (Tables 8 and 9).

Levene's test was used to inform the choice of appropriate post hoc tests for pairwise comparisons within commercial meats and within wild meats. Overall, pairwise comparisons revealed relatively few significant differences within commercial meats except for moisture and AA PC-3 (Table 11). By contrast, many significant pairwise comparisons were found within wild meats (Table 11).

PC-1 and PC-2 account for most of the variance (83.3%) in overall AA composition Table 6. Confidence ellipses in Figure 1 show distinct processed and wild meats in these two most important dimensions of AA-PC space. Distinguishable AA subgroups were also observed within wild meat (whitefish, pike, moose, mallard duck, and goose), but not within processed meats (Klik Light, submeats, chicken nuggets, hot dogs, and meatballs) (Figure 2). There is minimal overlap between wild and processed meat sources.

Multivariate ANOVA showed that processed meats were significantly distinguished from wild meats for both AA PC-1 and AA PC-2 scores (Table 7). MANOVA revealed significant differences within the wild meats for both AA PC-1 and AA PC-2 scores, but not within the processed meats (Table 11).

PC-1 was dominated by high positive loadings of most amino acids, particularly Tyr, Val, Ala, Thr, and Arg, and had a slight negative loading of OH-pro. AA PC-1 scores were significantly more positive in wild meats, indicating high levels of most amino acids, including Trp and other LNAAs (Tables 6 and 7). Processed meats tended to have low scores on AA-PC-1. Fish (whitefish and pike) samples had significantly lower AA PC-1 scores than mammal (moose) and waterfowl (mallard duck, and goose); and moose had a significantly lower AA PC-1 score than goose (Table 11).

PC-2 contrasted samples with positive loadings of Pro, OH-Pro, and Gly with strong negative loadings of Met, Lys, and Phe. Wild meats had significantly more negative scores on AA PC-2, associated with higher relative concentrations of Phe, Met, and Lys and lower Pro, OH-Pro, and Gly compared to processed meats. Goose tended to have the most positive scores on AA PC-2 and was significantly different than fish and moose (Table 11).

The absence of distinguishable ellipses among commercial meats (Figure 2) reflects the surprisingly high variability of AA composition between individual samples of a given commercial meat type. AA composition, including Trp, appears to be less variable within individual wild species than within single commercial meat products.

## 4. Discussion

### 4.1. Trp in Wild versus Processed Meat

Traditionally, the nomadic Cree subsisted on wild meat comparable to samples in the present study and ate very little carbohydrates. The selected processed meats in this study make up the largest commercial AA source in the modern Cree diet. We found significantly higher Trp in raw samples (ww) of wild game associated with significantly higher levels of protein and lower levels of “other” and fat (Table 7).

The units of g 100g^−1^ sample in our study represent the Trp available per serving of unprepared meat. The FAO recommends a mean Trp intake of 6.75mg/kg/d for “maintenance and growth” [34]. To obtain mean Trp requirements, the average Canadian (75g serving, 75kg person [44]) would require 2.8 daily servings of selected wild meat, compared to 3.8 daily servings of selected processed meat. Trp was highest in Canada goose, requiring 2.2 daily servings, and lowest in canned meatballs, requiring 4.5 daily servings.

Recall that dietary changes in Trp:LNAA proportionately alter blood plasma Trp:LNAA [22] and central 5-HT synthesis, due to LNAA competition for entry into the brain [45]. We found that the Trp:LNAA ratio was not statistically different between wild and processed meats (Table 5). Future work should consider, however, that relatively small fluctuations in Trp intake could have a large impact. First, the proportion of Trp in living organisms is very limited compared to other essential AAs and Trp levels throughout the body and brain must be tightly regulated to serve many critical functions [10]. We expected equal, or greater, regulation of AA proportions in commercial meat due to controlled and repetitive industrial procedures. Yet Figure 2 and Table 11 show high AA variability within processed types and insignificant differences between brands, unlike wild meats. Raw wild meats are more consistent and distinct within a given species.

Second, the amount of dietary Trp destined for central 5-HT is also limited. Only a small portion of Trp consumed is metabolized via the methoxyindole pathway for 5-HT synthesis due to competition with the more dominant kynurenine pathway, as well as protein synthesis, and alternative metabolites such as melatonin and tryptamine [10, 46, 47]. Most of this 5-HT pool is synthesized in the gut, reserving a small and controlled amount for the CNS [10, 48]. Thus, differential Trp availability between high-protein versus low-protein diets may disproportionately limit regulation of central 5-HT pools. Yet central 5-HT stores cannot be inferred from dietary Trp and LNAA content alone. Trp availability and transport for central 5-HT is also dictated by other factors: health conditions or medications causing Trp malabsorption [46]; excess Trp oxidation or metabolism [47, 49–51] changes in the kynurenine pathway [47, 52]; changes in peripheral 5-HT production [52, 53]; changes in gut microbiota [53]; chronic inflammation [54, 55]; and prolonged stress [51, 56, 57].

The introduction of this paper presents studies supporting Trp loss during industrial processing. There are several explanations for comparable proportions of Trp in wild and processed meat of our study. All processed samples in this study contained milk, soy, or fava bean protein that may be added after heat processing. Nielsen et al. have suggested that limited oxygen in stored foods may moderate potential Trp losses from processing [16]. Indeed, recent studies show significant heat-induced degradation of Trp in both meat and fish with home-cooking methods [58–60]. The very hydrophobic nature of Trp also places Trp residues deep inside proteins where they are more protected from degradation [9, 15, 18]. However, heat during industrial processing will denature proteins and may expose even deeply buried Trp to further heat treatments at home [61]. Heat also leads to isomerization of L-Trp to the D isomer, which is more difficult for humans to absorb and would go unrecognized by basic Trp quantification methods [15, 17, 35]. Heat can also alter protein digestibility and Trp availability, depending on the type of protein and heat treatment [29, 35, 62, 63]. Together, the current research leads us to predict that a greater degree of heat exposure, additives, mechanical modification, and chemical exposure will increase the risk of lowering plasma Trp:LNAA and central Trp availability; further analyses on AA exposure to variable treatments in food processing and preparation are needed.

Recently, Alipour [60] used standardized HPLC methods on Persian Sturgeon determined that frying led to a 95-99% Trp loss, but little change in other LNAA [60]. The LNAA content relative to the Trp content of fried sturgeon was 110 times higher than that of raw sturgeon (Table 12). Alternatively, Muszyńska et al. [64] observed a Trp increase of up to two orders of magnitude after heat treatment of some Basidiomycota mushroom varieties, a rise attributed to breakdown of larger indole compounds like 5-HT that were destroyed by thermal processing. Levels of 5-HT are significantly lower in a variety of heat-processed tomato products than fresh tomatoes [65]. It has not been determined if 5-HT is an important source of Trp in various foods.

### 4.2. Trp in Individual Meat Types of Present and Previous Studies

Despite the critical and diverse functionality of the nutrient Trp, the relatively recent elevation of Trp nutritional requirements, and the substantial improvements to Trp quantification methods, we do not have adequate Trp data for the human diet. Trp content in the U.S. Department of Agriculture's (USDA) Food Composition Database and Health Canada's Canadian Nutrient File is either absent, presented without reference, or obtained from outdated or unstandardized methods [66, 67]. Most often, Trp is estimated from gross mathematical constants (% of protein) that were determined before standardization.

The Canadian Food Inspection Agency (CFIA) estimates that Trp content is 1.5% in egg protein, 1.3% in milk, meat, poultry, or fish protein, and 1.1% from other or mixed protein sources [68]. Similarly, the USDA and Health Canada nutrient databases appear to infer total Trp content from the constant value of 1.12% Trp in fish protein and 1.39% in all skinless waterfowl protein, regardless of species or cooking treatments [66, 67]. We found pike and whitefish protein to have 0.92% and 0.98% Trp, respectively (Table 5), while Mohanty et al. [69] determined Trp to range from 0.2 to 6.5% of protein in twenty species of fish (Table 12). Trp analysis in fish demonstrates a much larger range of Trp content between species (Table 12) than what is inferred from constants used by Nutritional Databases.

Wild game in the present study had a mean of 1.12% Trp in moose and 1.26-1.27% Trp in waterfowl (Table 5). In a recent standardized analysis, Trp in protein was 0.99-1.20% for domestic goose, 0.70-1.25% in domestic duck (Table 13(a)), 0.94% in deer, and 0.60% in camel (Table 13(b)). Trp in waterfowl does not reach the 1.39% assumed by the USDA and Health Canada or the 1.3% assumed by the CFIA [66–68].

Trp is typically excluded from AA profiling of meat and thus comparable scientific studies are limited. The data that does exist suggests that the percentage of Trp in protein varies between different species and organisms. Trp analysis is most common in fish: our pike and whitefish data fell within the wide range of various fish species (Table 12). Moose Trp was slightly higher than deer and double that of camel (Table 13(b)). Canada goose and mallard duck Trp concentrations were also higher than those reported for domesticated Polish geese and pekin duck (Table 13(a)). Further, Trp levels in tissues within species, such as the pekin duck (Table 13(a)) and camel (Table 13(b)), are varied. The range of Trp:LNAA across our study meats is much narrower (more consistent) than findings from other literature on fish, domestic animals, and processed meat (Tables 12, 13(a), 13(b), and 13(c)); more analysis using standardized methods is needed.

Recommended Trp nutritional requirements highlight the substantial difference that can occur between actual Trp content and mathematical inferences. In the extreme example of the golden masheer (Table 12), experimentally measured Trp levels could provide daily Trp requirements with only a half serving, while mathematical inferences would lead to recommendations 8 times greater.

Processed meat protein in our study contained 1.03-1.27% Trp (Table 5). Recent AA profiles for processed meat are absent from the literature. When considering nutritional requirements, Trp content in these samples are comparable to the CFIA estimate of 1.11% for mixed protein sources. The USDA nutrient database provides measured Trp in some processed meats for the years 2002-2007 but gives no reference to specific studies or methods (Table 13(c)). Notably, the proportion of Trp in protein of our Klik Light (1.27%) was much higher than canned luncheon meat (0.98%) or bologna (0.79-1.09%) of the USDA, yet the proportion in our hot dogs (1.04%) was lower than USDA frankfurters (1.17%). Trp in our mixed submeats (1.03%) fell within the range of comparable USDA mixed meats (0.79-1.19%).

Overall, the estimation of total dietary Trp available in a given food type may not be accurately estimated from the existing assumption that Trp is a constant percentage in fish or meat protein. Fish and wild game of the present study and meat sources reported in previous studies have less Trp content than what is presented in major nutrient databases.

## 5. Conclusion

The dominant processed meats of the modern Cree diet are not equivalent substitutes for traditional meats in terms of total Trp content. Processed meats were significantly lower in Trp due to high proportions of nonprotein additives. Mallard ducks, Canada geese, and moose provide relatively high amounts of Trp and protein per serving when compared to wild fish and processed mixed meats. Trp composition closely paralleled protein content for all samples except fish, which had lower overall LNAA content. The proportion of Trp:LNAA in processed and wild meats was not significantly different. PCA revealed significant differences in AA composition between wild and processed meats: notably, essential dietary AAs (Trp, Tyr, Val, Thr, Met, Lys, and Phe) were more highly concentrated in wild meats.

Wild species of meat were more homogenous in composition than processed types, as well as in comparison to domesticated sources analyzed in other studies. Trp in our samples was higher than comparable samples in other studies, though many of the latter did not use standardized methods. Proportions of Trp in our fish and wild game protein were lower than estimated values assumed by CFIA, the USDA Food Composition Database, and the Canadian Nutrient File of Health Canada. These estimates do not provide accurate Trp composition of wild meat.

We summarized results from other Trp composition studies of comparable meat sources and identified a knowledge gap on Trp composition in traditional and modern diets. At present, Trp is the least quantified amino acid and thus composition studies using standardized methods are far too few to make general conclusions from interstudy comparisons. Trp is one of the most limited, labile, and multifunctional amino acids and is critical to mental wellness and general health. More research is needed to determine Trp content and AA proportions in the human diet, including unprepared foods and those subject to various cooking and preservation practices.

## Figures and Tables

**Table 1:** Processed Meat Collected for AA Analysis.

Type of Product	Replicates	Brand
Klik Light	3	Maple Leaf
Sub meats	3	Ezee Pizza Mix; Schneiders Extra Thick Bologna; Best Value Ham
Chicken Nuggets	3	Best Value, no breading
Hot Dog	3	Schneiders Red Hots
Meatballs	3	Puritan Meatballs in Gravy

**Table 2:** Wild Meat Collected for AA Analysis.

Meat Type	Replicates	Species
Northern Pike	5	*Esox lucius*
Whitefish	5	*Coregonus spp.*
Moose	5	*Alces alces*
Canada Goose	5	Branta canadensis
Mallard Duck	5	Anas platyrhynchos

**Table 3:** Proximate Composition of Processed and Wild Meats.

Proximate Composition (%) $\overset{-}{x}\pm SE$	Processed Meat^a^ (n=15)					Wild Meat^b^ (n=25)				
	Klik Light	Sub Meat	Chicken	Hot Dog	Meatballs	Pike	Whitefish	Moose	Mallard	Goose
Protein	14.92 ±2.15	17.83±1.03	15.44±1.42	15.76±4.00	12.34±1.46	20.16±0.86	18.43±0.62	21.84±0.87	23.61±1.08	24.78±0.49
Moisture	64.42±0.67	63.62±0.45	70.17±0.48	52.50±0.76	70.99±0.90	77.72 ±0.98	77.96±1.07	75.83±0.59	71.54±1.36	71.30±0.86
Fat	15.30±2.96	13.80±0.61	4.67±0.20	25.9±6.87	11.41±0.36	1.50±0.60	2.98±0.46	1.74±0.59	3.54±0.96	3.63±0.70
Other^c^	5.36±0.61	4.75±0.81	9.72±1.12	5.82±2.24	5.28±1.22	0.63±0.56	0.64±0.72	0.58±0.70	1.31±0.81	0.30±0.56

a. Means ± SE of 3 samples for each Processed Meat Type

b. Means ± SE of 5 samples for each Wild Meat Type

c. Other = carbohydrate, ash, sodium and preservatives not determined

**Table 4:** Total Tryptophan Content In Original Wet Samples Of Processed And Wild Meats.

Tryptophan (g•100g^−1^ sample) $\overset{-}{x}\pm SE$									
Processed Meat^a^ (n=15) 0.18 ± 0.02					Wild Meat^b^ (n=25) 0.24 ± 0.06				
Klik Light	Sub Meat	Chicken	Hot Dog	Meatballs	Pike	Whitefish	Moose	Mallard	Goose
0.19±0.03	0.19±0.02	0.19±0.03	0.16±0.02	0.15±0.02	0.19±0.01	0.18±0.01	0.24±0.03	0.29±0.01	0.31±0.06

a. Means ± SE of 3 samples for each Processed Meat Type

b. Means ± SE of 5 samples for each Wild Meat Type

**Table 5:** AA Composition of Protein in Processed and Wild Meats.

Amino Acid (g•100g^−1^ protein) $\overset{-}{x}\pm SE$	Processed Meat^a^ (Total n=15)					Wild Meat^b^ (Total n=25)				
	Klik Light	Sub Meat	Chicken	Hot Dog	Meatballs	Pike	Whitefish	Moose	Mallard	Goose
Tryptophan	1.27±0.18	1.03±0.08	1.27±0.05	1.04±0.19	1.18±0.04	0.92±0.04	0.98±0.07	1.12±0.17	1.26±0.06	1.27±0.16

Tyrosine	4.37±0.36	3.86±0.80	4.04±0.38	3.90±0.38	4.03±0.67	3.34 ±0.09	3.45± 0.09	3.69±0.09	3.75±0.24	3.82±0.45
Phenylalanine	3.84±0.25	3.51±0.14	4.00±0.26	3.70±0.07	4.30±0.15	4.40±0.17	4.35± 0.27	4.38±0.17	4.61±0.21	4.04±0.28
Valine	6.14±0.48	5.60±0.93	5.69±0.46	5.47±0.46	5.82±1.02	4.96±0.12	5.01±0.15	4.94±0.12	5.20±0.35	5.44± 0.60
Isoleucine	4.50±0.14	4.45±0.50	4.63±0.15	4.12±0.13	4.84±0.43	4.38±0.12	4.40±0.15	4.56±0.18	4.67±0.18	4.74±0.27
Leucine	8.02±0.22	7.46±0.78	7.60±0.21	7.25±0.09	7.96±0.73	7.45±0.11	7.33±0.24	7.89±0.15	8.12±0.36	8.05±0.50

Trp:LNAA	1:21	1:24	1:20	1:24	1:23	1:27	1:25	1:23	1:21	1:21

Histidine	3.40 ±0.35	2.77±0.68	3.35±0.42	3.23±0.67	3.14±0.77	2.05±0.04	2.18±0.08	2.50±0.10	2.55±0.23	2.80±0.42
Methionine	1.06 ±0.04	1.48±0.54	1.11±0.09	0.94±0.03	1.28±0.19	2.74±0.82	3.12±0.06	2.77±0.11	1.24±0.19	1.40±0.63
Lysine	5.75 ±0.95	5.59±0.61	5.69±0.70	6.56±0.21	6.72±0.31	9.54±0.63	9.56±0.89	9.21±0.53	8.92±0.68	7.44±1.17
Aspartic Acid	11.24 ±1.40	9.18±1.97	11.33±1.27	11.40±2.71	10.50±2.59	9.23±0.17	8.90±0.24	8.33±0.15	8.35±0.71	8.97±1.16
Glutamic Acid	14.50±5.40	11.92±2.21	13.23±1.70	19.69±4.68	11.04±2.01	13.32±0.22	12.97±0.70	14.03±0.33	13.27±1.10	14.21±1.68
Hydroxyproline	0.42±0.06	1.06±0.11	0.32±0.06	1.71±0.65	0.54±0.21	0.33±0.11	0.30±0.15	0.21±0.25	0.10±0.03	0.13±0.09
Serine	5.62±0.53	4.46±1.40	5.85±0.67	5.52±1.29	5.32±1.23	3.76±0.06	3.64±0.06	3.67±0.05	3.71±0.32	3.83±0.66
Glycine	5.07±0.52	5.21±1.16	4.73±0.60	6.45±1.56	4.88±1.25	3.80±0.31	3.86±0.41	3.32±0.40	3.44±0.28	3.54±0.51
Arginine	8.48±0.89	7.43±1.84	8.70±0.96	8.57±2.01	8.11±1.80	5.71±0.21	5.85±0.09	6.32±0.16	6.26±0.51	6.62±0.77
Threonine	5.33±0.55	4.53±1.29	5.10±0.64	5.20±1.09	5.02±1.33	3.92±0.10	4.17±0.14	4.21±0.13	4.32±0.35	4.51±0.68
Alanine	6.16±0.65	5.60±1.24	5.74±0.76	6.28±1.10	5.72±1.37	4.82±0.13	4.84±0.06	4.67±0.12	4.97±0.40	5.19±0.73
Proline	4.94±0.55	4.95±1.02	5.10±0.60	5.90±1.19	4.67±0.94	2.96±0.16	2.98±0.18	3.31±0.15	3.20±0.23	3.36±0.44
Alpha-Aminobutyric Acid	0.03±0.01	0.03±0.02	0.03±0.01	0.06±0.01	0.02±0.01	0.01±0.00	0.01±0.00	0.01±0.00	0.02±0.01	0.02±0.01

a. Means ± SE of 3 samples for each Processed Meat Type

b. Means ± SE of 5 samples for each Wild Meat Type

**Table 6:** Loadings of Amino Acids on Principal Components.

Amino Acid Concentration: log10 (1 + [g•100g^−1^ sample])	AA PC-1 (62.6%)	AA PC-2 (20.7%)	AA PC-3 (6.6%)
Tryptophan	*0.83*	-0.22	-0.24
Tyrosine	*0.99*	-0.06	-0.09
Valine	*0.98*	-0.08	-0.03
Isoleucine	*0.92*	-0.36	0.00
Leucine	*0.93*	-0.33	0.01
Histidine	*0.89*	0.32	-0.25
Phenylalanine	*0.76*	-0.58	0.12
Methionine	0.22	-0.73	0.50
Lysine	0.60	-0.70	0.31
Aspartic Acid	*0.93*	0.13	0.07
Glutamic Acid	*0.84*	-0.04	0.35
OH-Pro	-0.27	*0.70*	0.62
Serine	*0.76*	0.51	-0.23
Glycine	0.57	0.67	0.39
Arginine	*0.97*	0.12	-0.11
Threonine	*0.97*	0.12	-0.11
Alanine	*0.97*	0.17	0.05
Proline	0.56	*0.78*	0.04
Alpha-Aminobutyric Acid	0.32	0.55	0.16

**Table 7:** 1-Way ANOVAs Comparing Wild and Processed Meat.

Dependent Variable	F value	p value	Observed Power
Protein (g•100g^−1^ sample)	62.38	*< 0.005 *	1.00
Moisture (g•100g^−1^ sample)	44.25	*< 0.005 *	1.00
Fat (g•100g^−1^ sample)	55.56	*< 0.005 *	1.00
Other^a^ (g•100g^−1^ sample)	137.50	*< 0.005 *	1.00
Tryptophan (g•100g^−1^ sample)	17.43	*< 0.005 *	0.98
log10 (1 + Trp [g•100g^−1^ sample])	17.75	*< 0.005 *	0.98
Tryptophan:LNAA	0.76	0.39	0.14
Amino Acid PC-1 (62.6%)	19.46	*< 0.005 *	0.99
Amino Acid PC-2 (20.7%)	42.32	*< 0.005 *	1.00
Amino Acid PC-3 (6.6%)	1.82	0.19	0.26

a. Other = carbohydrate, ash, sodium and preservatives not determined

**Table 8:** 1-Way ANOVA Comparing Types of Processed Meat.

Dependent Variable	F-ratio	p-value	Observed Power
Protein (g•100g^−1^ sample)	2.256	0.135	0.454
Moisture (g•100g^−1^ sample)	363.984	*<0.001*	1.000
Fat (g•100g^−1^ sample)	15.782	*<0.001*	1.000
Other (g•100g^−1^ sample)	6.889	*0.006*	0.930
Tryptophan (g•100g^−1^ sample)	2.650	0.096	0.524
log10 (1 + Trp [g•100g^−1^ sample])	2.396	0.120	0.479
Tryptophan:LNAA	0.683	0.619	0.157
Amino Acid PC-1 (62.6%)	1.854	0.195	0.379
Amino Acid PC-2 (20.7%)	2.593	0.101	0.514
Amino Acid PC-3 (6.6%)	14.806	*<0.001*	0.999

**Table 9:** 1-Way ANOVA Comparing Types of Wild Meat.

Dependent Variable	F-ratio	p-value	Observed Power
Protein (g•100g^−1^ sample)	50.007	*<0.001*	1.000
Moisture (g•100g^−1^ sample)	52.536	*<0.001*	1.000
Fat (g•100g^−1^ sample)	10.752	*<0.001*	0.999
Other (g•100g^−1^ sample)	1.531	0.231	0.387
Tryptophan (g•100g^−1^ sample)	9.482	*<0.001*	0.997
log10 (1 + Trp [g•100g^−1^ sample])	34.964	*<0.001*	1.000
Tryptophan:LNAA	5.952	*0.003*	0.952
Amino Acid PC-1 (62.6%)	38.100	*<0.001*	1.000
Amino Acid PC-2 (20.7%)	8.632	*<0.001*	0.994
Amino Acid PC-3 (6.6%)	7.013	*0.001*	0.978

**Table 10:** Ingredients List of Processed Meats in the Present Study.

Meat Product^a^	Ingredients
Klik Light “Canadian Spam”	Pork, mechanically separated pork, water, modified corn starch, soy protein product, sodium erythorbate, sodium nitrite

Sub Meats	
Best Value Ham	Pork, water, glucose solids and/or dextrose and/or sugar, potassium lactate, salt, potassium chloride, sodium phosphate, flavour, carrageenan, sodium diacetate, sodium erythorbate, sodium bicarbonate, sodium nitrite, spices, smoke flavour
Extra Thick Bologna	Pork, chicken, water, wheat flour, salt, milk ingredients, potassium lactate, sodium erythorbate, sodium diacetate, sodium nitrite, garlic powder, spice, smoke
Maple Leaf Ezee Pizza	Pork, mechanically separated meat (chicken, pork), beef; water, wheat flour, salt, potassium lactate, sodium diacetate, dried garlic, sodium erythorbate, spice, flavour, potato starch, spice extract, sodium nitrite, smoke, potassium pyrophosphate, dextrose, carraggeenan

Chicken Nugget	Chicken, water, toasted wheat crumbs, textured soy protein, modified cornstarch, wheat flour, yellow corn flour, modified potato starch, soy protein, wheat starch, salt, onion powder, baking powder, canola, and/or sunflower and/or palm and/or palm kernel oil, shortening, dried egg powder (contains baker's yeast, citric acid), modified milk ingredients, corn starch powder, defatted soy flour, potato maltodextrin, corn dextrin, guar gum, spices, browned egg in canola oil

Schneiders Red Hots	Pork, water, modified corn starch, skim milk powder, salt, potassium lactate, sodium erythorbate, sodium diacetate, sugar, sodium nitrite, wheat flour, spice extractives, garlic powder, smoke

Puritan Meatballs and Gravy	Formed meatballs (mechanically separated chicken, beef, toasted wheat crumbs, fava bean protein, salt), water, enriched wheat flour, modified corn starch, glucose-fructose, salt, monosodium glutamate, caramel colour, spice extracts

a. Note that right-from-the-package processed meats are typically exposed to heat, additives, mechanical manipulation and sterilization chemicals during the manufacturing process, and further heat processing prior to consumption.

**Table 11:** Pair-Wise Post Hoc Comparisons of Food Types.

*Food Source*	Dependent Variable^a^	(I) Food Type	(J) Food Type	Mean Difference (I-J)^b^	p-value^c^
*Processed*	Moisture (g•100g^−1^ sample)	Klik Light	Chicken Nugget	-5.743	< 0.001
		Klik Light	Hot Dog	11.923	< 0.001
		Klik Light	Meatballs	-6.540	< 0.001
		Sub Meats	Chicken Nugget	-6.547	< 0.001
		Sub Meats	Hot Dog	11.120	< 0.001
		Sub Meats	Meatballs	-7.343	< 0.001
		Chicken Nugget	Hot Dog	17.667	< 0.001
		Hot Dog	Meatballs	-18.463	< 0.001
	Fat (g•100g^−1^ sample)	Sub Meats	Chicken Nugget	9.133	0.006
		Chicken Nugget	Meatballs	-6.740	0.001
	Other (g•100g^−1^ sample)	Klik Light	Chicken Nugget	-4.367	0.024
		Sub Meats	Chicken Nugget	-4.973	0.010
		Chicken Nugget	Hot Dog	3.900	0.049
		Chicken Nugget	Meatballs	4.440	0.022
	Amino Acid PC-3 (6.6%)	Klik Light	Sub Meats	-1.820	0.035
		Klik Light	Hot Dog	-2.632	0.003
		Sub Meats	Chicken Nugget	1.996	0.019
		Sub Meats	Meatballs	1.914	0.025
		Chicken Nugget	Hot Dog	-2.809	0.002
		Hot Dog	Meatballs	2.726	0.002

*Wild *	Protein (g•100g^−1^ sample)	Pike	Whitefish	1.722	0.031
		Pike	Moose	-1.684	0.036
		Pike	Mallard	-3.450	< 0.001
		Pike	Goose	-4.620	< 0.001
		Whitefish	Moose	-3.406	< 0.001
		Whitefish	Mallard	-5.172	< 0.001
		Whitefish	Goose	-6.342	< 0.001
		Moose	Mallard	-1.766	0.025
		Moose	Goose	-2.936	< 0.001
	Moisture (g•100g^−1^ sample)	Pike	Mallard	6.182	< 0.001
		Pike	Goose	6.428	< 0.001
		Whitefish	Moose	2.118	0.033
		Whitefish	Mallard	6.412	< 0.001
		Whitefish	Goose	6.658	< 0.001
		Moose	Mallard	4.294	< 0.001
		Moose	Goose	4.540	< 0.001
	Fat (g•100g^−1^ sample)	Pike	Whitefish	-1.482	0.027
		Pike	Mallard	-2.046	0.001
		Pike	Goose	-2.138	0.001
		Moose	Mallard	-1.798	0.005
		Moose	Goose	-1.890	0.003
	Tryptophan (g•100g^−1^ sample)	Pike	Mallard	-0.336	< 0.001
		Whitefish	Mallard	-0.280	0.001
	log10 (1 + Trp [g•100g^−1^ sample])	Pike	Mallard	-0.039	< 0.001
		Pike	Goose	-0.044	0.007
		Whitefish	Mallard	-0.042	< 0.001
		Whitefish	Goose	-0.047	0.003
	Tryptophan:LNAA	Pike	Mallard	-0.010	0.015
		Pike	Goose	-0.011	0.011
		Whitefish	Goose	-0.009	0.049
	Amino Acid PC-1 (62.6%)	Pike	Moose	-0.622	0.043
		Pike	Mallard	-1.212	< 0.001
		Pike	Goose	-1.659	< 0.001
		Whitefish	Moose	-1.016	< 0.001
		Whitefish	Mallard	-1.606	< 0.001
		Whitefish	Goose	-2.053	< 0.001
		Moose	Goose	-1.037	< 0.001
	Amino Acid PC-2 (20.7%)	Pike	Goose	-0.933	0.003
		Whitefish	Goose	-1.089	0.001
		Moose	Goose	-0.960	0.003
	Amino Acid PC-3 (6.6%)	Pike	Mallard	1.380	0.005
		Pike	Goose	1.339	0.007
		Whitefish	Mallard	1.136	0.029
		Whitefish	Goose	1.096	0.038

a. Only variables determined to have significant differences between food-type (Tables 8 and 9) were examined in pairwise comparisons

b. Based on observed means

c. Bonferroni or Tamhane's T2 post-hoc test, as appropriate for variance heterogeneity

**Table 12:** Tryptophan Content in Fish of Present Study and Previous Studies.

Common Name	Scientific Name	Protein (g•100g^−1^ sample)	Trp (g•100g^−1^ sample)	Trp (g•100g^−1^ protein)	Trp:LNAA
Northern pike^a^	*Esox lucius*	20.16	0.19	0.92	1:27
Whitefish^a^	*Coregonus spp.*	18.43	0.18	0.98	1:25
Walking catfish^b^	*Clarias batrachus*	16.40	0.18	1.10	1:22
Asian stinging catfish^^b^^	*Heteropneustes fossilis*	16.30	0.10	0.60	1:45
Giant river-catfish^b^	*Sperata seenghala*	19.00	0.04	0.20	nd
Rainbow trout ^b^	*Oncorhynchus mykiss*	17.20	1.07	6.20	1:5
Yellowfin tuna^b^	*Thunnus albacores*	23.90	0.38	1.60	1:16
Yellowfin tuna^c^	*Thunnus albacares*	23.52	0.23	0.99	1:25
Bigeye tuna^c^	*Thunnus obesus*	23.72	0.23	0.96	1: 26
Persian sturgeon, raw^d^	*Acipenser persicus*	21.40	0.24	1.11	1:24
Persian sturgeon, grilled^d^	*Acipenser persicus*	31.00	0.02	0.05	1:528
Persian sturgeon, fried^d^	*Acipenser persicus*	32.00	0.00	0.01	1:2645
Atlantic halibut^e^	*Hippoglossus hippoglossus*	nd	nd	1.07	1:23
Yellowtail flounder^e^	*Pleuronectes ferruginea*	nd	nd	1.32	1:18
Japanese flounder^e^	*Paralichthys olivaceus*	nd	nd	1.06	1:23
Common snowtrout^b^	*Schizothorax richardsonii*	16.30	0.07	0.40	nd
Mola^b^	*Amblypharyngodon*	16.30	0.03	0.20	Nd
Climbing perch^b^	*Anabas testudineus*	16.90	0.24	1.40	1:20
Major carp^b^	*Catla catla*	16.20	0.16	1.00	1:26
Mrigal carp^b^	*Cirrhinus mrigala*	15.50	0.09	0.60	1:46
Common carp^b^	*Cyprinus carpio*	17.20	0.15	0.90	1:6
Rohu carp^b^	*Labeo rohita*	15.90	0.08	0.50	1:56
Japanese threadfin bream^b^	*Nemipterus japonicus*	15.40	0.35	2.30	1:11
Pool barb^b^	*Puntius sophore*	16.30	0.02	0.10	Nd
Indian mackerel^b^	*Rastrelliger kanagurta*	19.20	0.23	1.20	1:23
Commerson's anchovy^b^	*Stolephorus commersonii*	16.40	0.34	2.10	1:11
Spotty-face anchovy^b^	*Stolephorus waitei*	20.30	0.43	2.10	1:12
Ilish (Herring)^b^	*Tenualosa ilisha*	20.70	0.04	0.20	Nd
Copper masheer^b^	*Neolissochilus hexagonolepis*	18.20	0.07	0.40	1:17
Golden masheer^b^	*Tor putitora*	17.00	1.11	6.50	1:4

a. Present study (fish samples were skinless and from the fillet portion)

b. Mohanty et al. 2014 (NaOH, spectrophotometry) [69]

c. Peng et al., 2013 (alkaline, ion exchange chromatography) [70]

d. Alipour et al. 2010 (NaOH, HPLC) [60]

e. Kim and Lall, 2000 (alkaline, colorimetric) [71]

**Table:** (a) Tryptophan Content in Waterfowl of Present Study and Previous Studies.

Common Name	Scientific Name or Domestic Variety	Protein (g•100g^−1^ sample)	Trp (g•100g^−1^ sample)	Trp (g•100g^−1^ protein)	Trp:LNAA
Canada Goose^a^	*Branta canadensis*	24.78	0.31	1.27	1:21
Domestic Goose, breast^b^	Garbonosa	21.96	0.24	1.11	1:22
Domestic Goose, thigh^b^	Garbonosa	21.36	0.25	1.18	1:22
Domestic Goose, breast^b^	Rypinska	21.82	0.22	0.99	1:29
Domestic Goose, thigh^b^	Rypinska	21.17	0.25	1.20	1:25
Mallard Duck^a^	*Anas platyrhynchos*	23.61	0.29	1.26	1:21
Domestic Duck, thigh^c^	Pekin: 4 varieties	20.44-20.66	0.16-0.25	0.77-1.19	1:24
Domestic Duck, breast^d^	Pekin: 5 varieties	19.53-28.77	0.15-0.24	0.70-1.25	1:26

a. Present study (birds samples were skinless and from the pectoral muscle)

b. Okruszek et al. 2013 (Ba(OH)_2_, HPLC) [72]

c. Woloszyn et al. 2011 (Ba(OH)_2_, HPLC) [40]

d. Woloszyn et al. 2006 (Ba(OH)_2_, HPLC) [39]

**Table:** (b) Tryptophan Content in Ungulates of the Present Study and Previous Studies.

Common Name	Scientific Name	Protein (g•100g^−1^ sample)	Trp (g•100g^−1^ sample)	Trp (g•100g^−1^ protein)	Trp:LNAA
Moose^a^	*Alces alces*	21.84	0.24	1.12	1:23
Camel, various cuts averaged^b^	*Camelus dromedarius*	19.88	0.12	0.60	1:44
Camel, liver^b^	*Camelus dromedarius*	20.76	0.27	1.30	1:21
Camel, heart^b^	*Camelus dromedarius*	16.79	0.13	0.75	1:36
Camel, kidneys^b^	*Camelus dromedarius*	15.01	0.16	1.07	1:24
Deer^c^	*Cervus elaphus maral*	18.71	0.18	0.94	nd

a. Present study (moose samples were skinless and from the hindquarter)

b. Dawood and Alkanhal, 1995 (unknown, colorometric) [37]

c. Okuskhanova et al. 2017 (organic acid, HPLC) [73]

**Table:** (c) Tryptophan Content in Processed Meat of Present Study and Reported by USDA.

Food Type	Protein (g•100g^−1^ sample)	Trp (g•100g^−1^ sample)	Trp (g•100g^−1^ protein)	Trp:LNAA
Chicken nugget, unbreaded, uncooked^a^	15.44	0.19	1.27	1:21
Chicken, commodity, canned, drained^c^	27.52	0.32	1.17	1:21
Meatball^a^	12.34	0.15	1.18	1:23
Sub Meat: pork, chicken, beef^a^	17.83	0.19	1.03	1:24
Bologna, chicken and pork^b^	10.31	0.08	0.79	1:31
Bologna, pork, turkey, beef^b^	11.56	0.13	1.09	1:23
Pepperoni, beef and pork, sliced^b^	19.25	0.23	1.19	1:21
Hot Dog, (pork)^a^	15.76	0.16	1.04	1:24
Frankfurter (pork)^b^	12.81	0.15	1.17	1:21
Klik Light (pork)^a^	14.92	0.19	1.27	1:21
Luncheon meat, pork, canned^b^	12.50	0.12	0.98	1:25

a. Present study (processed meats were right-from-the-package prior to home cooking and consumption)

b. USDA 1980-2007 (unknown methods and sources) [66]

## Data Availability

The data used to support the findings of this study are included within the article.

## Abbreviations

AA: Amino Acid
Trp: Tryptophan
LNAA: Large neutral amino acid
UPLC: Ultra performance liquid chromatography
USDA: United States Department of Agriculture
CFIA: Canadian Food Inspection Agency
FAO: Food and Agricultural Organization
(M)ANOVA: (Multivariate) Analysis of variance
Tyr: Tyrosine
Val: Valine
Ala: Alanine
Thr: Threonine
Arg: Arginine
OH-pro: Hydroxyproline
Pro: Proline
Gly: Glycine
Met: Methionine
Lys: Lysine
Phe: Phenylalanine.
